# A bibliometric analysis of natural language processing in medical research

**DOI:** 10.1186/s12911-018-0594-x

**Published:** 2018-03-22

**Authors:** Xieling Chen, Haoran Xie, Fu Lee Wang, Ziqing Liu, Juan Xu, Tianyong Hao

**Affiliations:** 1 College of Economics, Jinan University, Guangzhou, China; 2 Department of Mathematics and Information Technology, The Education University of Hong Kong, Hong Kong, Hong Kong, Special Administrative Region of China; 3 School of Science and Technology, The Open University of Hong Kong, Hong Kong, Hong Kong, Special Administrative Region of China; 4 The Second Clinical Medical College, Guangzhou University of Chinese Medicine, Guangzhou, China; 5 The Research Institute of National Supervision and Audit Law, Nanjing Audit University, Nanjing, China; 6 School of Information Science and Technology, Guangdong University of Foreign Studies, Guangzhou, China; 7 School of Computer, South China Normal University, Guangzhou, China

**Keywords:** Natural language processing, Medical, Bibliometrics, Statistical characteristics, Scientific collaboration, Thematic discovery and evolution

## Abstract

**Background:**

Natural language processing (NLP) has become an increasingly significant role in advancing medicine. Rich research achievements of NLP methods and applications for medical information processing are available. It is of great significance to conduct a deep analysis to understand the recent development of NLP-empowered medical research field. However, limited study examining the research status of this field could be found. Therefore, this study aims to quantitatively assess the academic output of NLP in medical research field.

**Methods:**

We conducted a bibliometric analysis on NLP-empowered medical research publications retrieved from PubMed in the period 2007–2016. The analysis focused on three aspects. Firstly, the literature distribution characteristics were obtained with a statistics analysis method. Secondly, a network analysis method was used to reveal scientific collaboration relations. Finally, thematic discovery and evolution was reflected using an affinity propagation clustering method.

**Results:**

There were 1405 NLP-empowered medical research publications published during the 10 years with an average annual growth rate of 18.39%. 10 most productive publication sources together contributed more than 50% of the total publications. The USA had the highest number of publications. A moderately significant correlation between country’s publications and GDP per capita was revealed. *Denny, Joshua C* was the most productive author. *Mayo Clinic* was the most productive affiliation. The annual co-affiliation and co-country rates reached 64.04% and 15.79% in 2016, respectively. 10 main great thematic areas were identified including *Computational biology*, *Terminology mining*, *Information extraction*, *Text classification*, *Social medium as data source*, *Information retrieval*, etc.

**Conclusions:**

A bibliometric analysis of NLP-empowered medical research publications for uncovering the recent research status is presented. The results can assist relevant researchers, especially newcomers in understanding the research development systematically, seeking scientific cooperation partners, optimizing research topic choices and monitoring new scientific or technological activities.

## Background

Natural language processing (NLP) is a theoretically motivated range of computational techniques for the automatic analysis and representation of human language [1]. Its goal is to realize human-like language understanding for a wide range of applications and tasks [2]. As a large and complex domain, medicine is rich in synonymy and semantically similar and related concepts [3]. Most clinical information resources including Electronic Medical Records (EMRs), Electronic Health Records (EHRs) and medical knowledge contain considerable amount of information. However, much of this information comes in unstructured form, also called free-text [4]. NLP is crucial for transforming relevant unstructured information hidden in free-text into structured information and is extremely useful in improving healthcare and advancing medicine [5].

There have been rich research achievements of NLP methods and applications for processing medical information [6]. Emerging interests of medical information processing with NLP methods include speech information recognition [7], semantic labeling [8], syntactic parsing [9], word sense disambiguation [10, 11], negation detection [12], and temporal analysis [13, 14]. Medical practical problems can also gain solutions from NLP-empowered applications including adverse drug reactions detection [15], medication discrepancy detection [16], EMRs or EHRs coding and classification [17], clinical trial computation [18–21], etc. NLP-empowered medical research field grows fast and draws more and more attention [6]. It is of great significance to understand its research status through a systematic analysis on relevant research output.

In the analysis research, bibliometrics is defined as the use of statistical methods for quantitative assessment of academic output [22, 23]. Benefits of bibliometric analysis include evaluating leading scientific researchers or publications [24], studying the structure of the network of a scientific field [25], identifying major topics [26], discovering new developments [27], etc.

This paper thus carries out a thorough bibliometric analysis on NLP-empowered medical publications from PubMed during the year 2007–2016. The descriptive statistics analysis, social network analysis, and Affinity Propagation clustering analysis are used in the analysis. Specifically, the purpose of the analysis is to: 1) identify productive publication sources, authors, affiliations, and countries in NLP-empowered medical research field; 2) visualize the number of countries and scientific collaboration among authors and affiliations; and 3) distinguish major themes and their evolution.

## Related work

Applications of bibliometrics are numerous. Many studies focused on publication statistical characteristics evaluation with elements such as publication data, influential journals, productive authors, affiliations, and countries. Based on two separate databases Web of Science (WoS) and Google Scholar, Diem and Stefan [28] investigated the fitness-for-purpose of bibliometric indicators for measuring the research performance of individual researchers in education sciences field in Switzerland. The study results indicated that the indicators for research performance measurement such as quantity of publications and citation impact measure were highly positively correlated. Fan et al. [29] conducted a bibliometric study for the evaluation of the quantity and quality of Chinese publications on burns at both the international and domestic levels with basis of PubMed records during 1985 and 2014. Similar works have also been conducted for medical research output. A study for the determination of whether a correlation existed between bibliometrics and National Institutes of Health (NIH) funding data among academic neurosurgeons was conducted by Venable et al. [30]. Their work revealed that bibliometric indices were higher among neurosurgeons with NIH funding, but only the contemporary h-index was shown to be predictive of NIH funding. By examining the growth of published literature on diabetes in three countries including Nigeria, Argentina and Thailand, Harande and Alhaji [31] showed that the literature of the disease grew and spread very widely. Ramos [32] found that the research output in countries with more estimated cases of tuberculosis was less compared with industrialized countries through a bibliometric analysis of tuberculosis research output. In addition, bibliometric analysis on research publications related with cancer [33], eye disease [34], obesity [35], dental traumatology [36], etc., could also be found. Bibliometric analysis for publication statistical characteristics evaluation was also available for specific journals, e.g., *Journal of Intellectual Property Rights* [37] and *The Electronic Library* [38].

Studies on collaboration relationship among authors, affiliations, or countries were commonly found. Based on researches covering biomedical, physics, and mathematics, Newman [39] compared the scientific co-authorship patterns using network analysis. Radev et al. [40] investigated the publications published by *The Association for Computational Linguistics* using citation and collaboration network analysis to identify the most central papers and authors. A bibliometric and visual study on consumer behavior research publications from 1966 to 2008 was presented by Muñoz-Leiva et al. [41]. Geaney et al. [42] provided a detailed evaluation of type 2 diabetes mellitus research output during the year 1951–2012 with methods of large-scale data analysis, bibliometric indicators, and density-equalizing mapping. They came to the conclusion that the number of research was rising in step with the increasing global burden of the disease. With a chord diagram of the 20 most productive countries, Li et al. [43] confirmed the predominance of the USA in international geo-ontology research collaboration. They also found that the international cooperation of countries such as Sweden, Switzerland, and New Zealand were relatively high although with fewer publications.

There were also a few studies centering on research topic detection of a certain field using bibliometrics. For example, Heo et al. [44] analyzed the field of bioinformatics using a multi-faceted topic modeling method. By combining performance analysis and science mapping, some studies conducted thematic evolution detection and visualization of a given research field, e.g., hydropower [45], neuroscience [46], and social work [47]. Similar works have also been conducted for specific journals such as *Knowledge-Based Systems* [22]. Based on co-word analysis, Cobo et al. [48] proposed an automatic approach with the combination of performance analysis and science mapping to show the conceptual evolution of intelligent transportation systems research field during three consecutive periods. Six main thematic areas were identified out. With the purpose of mapping and analyzing the structure and evolution of the scientific literature on gender differences in higher education and science, Dehdarirad et al. [49] applied co-word analysis to identify main concepts, used hierarchical cluster analysis to cluster the keywords, and created a strategic diagram to analyze trends.

Most relevant studies chose WoS as publication retrieval data source, and therefore, author-defined keywords and ISI keywords plus were usually used as topic candidates [22, 23, 46]. This might lead to information loss without considering title and abstract fields. The key terms in title and abstract fields were extracted and analyzed using VOSviewer with equal importance in the study of Yeung et al. [46]. However, it is more reasonable to bestow weighing for terms from different fields.

To our knowledge, there was no study applying bibliometrics to assess research output of NLP-empowered medical research field. Therefore, giving the deficiencies in existing research, this study uses PubMed as data source. With 1405 NLP-empowered medical research publications retrieved, literature distribution characteristics and scientific collaboration are acquired using a descriptive statistics method and a social network analysis method, respectively. In addition to author defined keywords and PubMed medical subject headings (MeSH), key terms extracted from title and abstract fields using a developed Python program are also included in AP clustering analysis for thematic discovery and evolution.

## Methods

### Data set

PubMed is an important data source on life sciences and biomedical topics. We used PubMed as data source and downloaded documents using the following query: ((“2007”[Publication Date]: “2016”[Publication Date])) AND ((“NLP”[Title] OR “Natural Language Processing”[Title]) OR (“NLP”[Title/Abstract] OR “Natural Language Processing”[Title/Abstract])).

Using the query, we retrieved a total of 1776 documents in XML format. Key elements including title, published year, publication source, author address, author keywords, PubMed MeSH, and abstract were extracted. Due to the issues of information missing and irrelevant documents, manual information supplement and document exclusion were conducted. After that, 1405 NLP-empowered medical research publications between 2007 and 2016 were identified out as dataset.

According to the author addresses information, the corresponding affiliations and countries were manually preprocessed and automated identified. As for the title and abstract fields, a developed Python program was applied to extract key terms (including single words and phrases). According to observation on 50 samples, we found that most of the extracted single words were meaningful, e.g., “influenza”, “surveillance”, and “misdiagnoses”. Furthermore, in order to improve data quality, a de-duplicating process was applied (author defined keywords, PubMed MeSH, and extracted key terms as units of analysis). Some abbreviations were replaced by the corresponding full-names. For example, “EHR” was replaced by “Electronic Health Record”. Words representing the same concepts were grouped. In addition, words with a very broad and general meaning, e.g., “natural language processing”, “algorithm”, were removed. After the above pre-processing, the dataset was analyzed using software R. Some statistical characteristics of the dataset are shown as Table 1.

**Table 1:** The statistical characteristics of the dataset

Characteristics	Statistics
Total #pub.	1405
#pub. with author address information	1386
#pub. with abstract	1382
#pub. with author keywords or PubMed MeSH	1277
#unique publication sources	324
#unique countries/first countries	56/45
#unique authors/first authors	4391/1053
#unique affiliations/first affiliations	961/514
Average #words/word characters in title	12.53; 6.50
Average number/standard deviation of character in title	95.43; 29.72
Average #words/word characters in abstract	215.24; 5.62
Average number/standard deviation of character in abstract	1456.95; 536.2
Top 10 frequency words/phrases in author keywords or PubMed MeSH	Electronic health record (363; 25.84%); Data mining (278; 19.79%); Information storage and retrieval (239; 17.01%); Artificial intelligence (179; 12.74%); Female (163; 11.60%); Semantics (156; 11.10%); Male (153; 10.89%); Controlled vocabulary (140; 9.96%); Automatic pattern recognition (127; 9.04%); Medical record system (112; 7.97%)
Top 10 frequency words/phrases extracted from title	Electronic health record (69; 4.91%); Medical record (55; 3.91%); Clinical text (45; 3.20%); Clinical note (41; 2.92%); Patient (37; 2.63%); Text mining (23; 1.64%); Classification (22; 1.57%); Clinical narrative (21; 1.49%); Radiology report (21; 1.49%); Natural language processing method (20; 1.42%)
Top 10 frequency words/phrases extracted from abstract	Patient (322; 22.92%); Precision (217; 15.44%); F-measure (205; 14.59%); Recall (178; 12.67%); Accuracy (164; 11.67%); Electronic health record (161; 11.46%); Natural language processing method (155; 11.03%); Medical record (143; 10.18%); Disease (141; 10.04%); Concept (128; 9.11%)

### Statistical analysis

Some publication characteristics are obtained through statistical analysis using a descriptive statistical method, a regression analysis method, and a hypothesis testing method. Descriptive statistics is used for quantitatively summarizing the basic characteristics of a collection of data [50]. It simplifies large amounts of data in a sensible way by presenting quantitative descriptions in a manageable form, generally along with simple graphics analysis. Regression analysis is a set of statistical processes for estimating the relationships between a dependent variable and one or more independent variables. It helps one find out how the dependent variable changes when any one of the independent variables is varied while the other independent variables remain fixed. As a method of statistical inference, statistical hypothesis testing is used to determine whether a hypothesis is a reasonable statement and should not be rejected or if it is an unreasonable statement and should be rejected based on sample statistics and probability theory. A hypothesis is proposed for the statistical relationship between two datasets as an alternative, and is compared with an idealized null hypothesis proposing no relationship between two datasets. The comparison is regarded statistically significant if the null hypothesis is unlikely to realize according to a threshold probability, i.e., a significance level.

In this study, a descriptive statistics method was applied to acquire the distribution characteristics of the dataset, including publication distribution by year, productive publication sources, authors, affiliations, and countries, as well as annual cooperation publication distribution. Based on the number of publications, 3 fitting models including linear model without intercept, linear model with intercept, and non-linear model with quadratic term, were built with *year/1000* and (*year/1000)*^*2*^ as independent variables. Akaike Information Criterion (AIC) and adjusted R-squared ($\overline{R^2}$) were used to select the optimal fitting model. In order to understand the relationship between number of publications and GDP per capita, a Spearman’s rank correlation test was applied to test the hypothesis as:

### Hypothesis: There is no significant relationship between publications and GDP per capita

Spearman’s rank correlation coefficient is a nonparametric measure of statistical dependence between the rankings of two variables, which is expressed as:

1 $$ {r}_s={\rho}_{r{g}_X,r{g}_Y}=\frac{\operatorname{cov}\left(r{g}_X,r{g}_Y\right)}{\sigma_{r{g}_X}{\sigma}_{r{g}_Y}} $$

where *ρ* denotes the usual Pearson correlation coefficient, but applies to the rank variables. cov(*rg*_*X*_, *rg*_*Y*_) is the covariance of the rank variables. ${\sigma}_{r{g}_X}$and ${\sigma}_{r{g}_Y}$are the standard deviations of rank variables.

### Geographic visualization

Geographic visualization is a set of techniques for analyzing spatial data with an emphasis on knowledge construction over knowledge storage or information transmission. Aiming at facilitating the exploration, analysis, synthesis, and presentation of georeferenced information, geographic visualization integrates principles from geographic information systems, exploratory data analysis, cartography, as well as information visualization [51].Techniques such as multimedia, image processing, computer graphics, and virtual reality are combined for presenting information in a way that patterns can be found, and greater understanding can be acquired. In this study, we applied geographic visualization analysis to explore worldwide geographical distribution of NLP-empowered medical research publications in country-level.

### Social network analysis

Social network analysis, related to network theory, is a process of investigating social structures based on networks and graph theory in modern sociology [52]. Social network perspective concentrates on relationships among social entities [53] with two main focuses, i.e., the actors and the relationships between them in a specific social context [54]. Networked structures are characterized in terms of nodes with the ties, edges, or links connecting them.

In this study, we applied social network analysis to explore the cooperation relationships for specific authors and affiliations in NLP-empowered medical research field. The cooperation among affiliations and authors were visualized with force directed network graphs, respectively. In the network graphs, the nodes represented specific affiliations or authors, and the lines represented the cooperation relationship. The size of node indicated the number of publications of a specific author or affiliation. The width of link indicated the cooperation frequencies between the two affiliations or authors.

### Term importance weighting

In thematic evolution discovery, author defined keywords, PubMed MeSH, and key terms extracted from title and abstract were jointly used as units of analysis. Since the importance of different parts of a publication was different, we conducted a weighting process with the combination of subjective and objective methods. Suppose there were *n* unique words among author defined keywords, PubMed MeSH, and key terms extracted from title and abstract of a sample of 30 publications (*p* = 1,2,…,30). The objective method was as Eq. (2).

2 $$ \Big\{{\displaystyle \begin{array}{c}0\le \alpha \le 1, stepsize=0.1\\ {}0\le \beta \le 1-\alpha, stepsize=0.1\\ {}\gamma =1-\alpha -\beta \\ {}{F}_{w_i,\alpha, \beta, \gamma}^O=\alpha {f}_{1{w}_i}+\beta {f}_{2{w}_i}+\gamma {f}_{3{w}_i},i=1,2,\dots, n\\ {}{F}_{\alpha, \beta, \gamma}^O=\left\{{F}_{w_i,\alpha, \beta, \gamma}^O,i=1,2,\dots, n\right\}\\ {}{R}_{\alpha, \beta, \gamma}^O=\mathit{\operatorname{rank}}\left({F}_{\alpha, \beta, \gamma}^O\right)\\ {}{R}_{\alpha, \beta, \gamma}^O=\left\{{R}_{w_i,\alpha, \beta, \gamma}^O,i=1,2,\dots, n\right\}\end{array}} $$

where *α*, *β*, and *γ* represented weights for author defined keywords and PubMed MeSH, key terms extracted from title, and key terms extracted from abstract, respectively. ${f}_{1{w}_i}$,${f}_{2{w}_i}$, and ${f}_{3{w}_i}$ represented the frequencies of word *w*_*i*_ in author defined keywords and PubMed MeSH, key terms extracted from title, and key terms extracted from abstract, respectively. ${F}_{w_i,\alpha, \beta, \gamma}^O$ was the frequency of word *w*_*i*_ weighted by *α*, *β*, and *γ*. ${F}_{\alpha, \beta, \gamma}^O$ was the mathematical set of ${F}_{w_i,\alpha, \beta, \gamma}^O$. ${R}_{\alpha, \beta, \gamma}^O$ was the objective ranking of${F}_{\alpha, \beta, \gamma}^O$.${R}_{w_i,\alpha, \beta, \gamma}^O$ was the ranking of word *w*_*i*_, and thus ${R}_{\alpha, \beta, \gamma}^O$ was the mathematical set of ${R}_{w_i,\alpha, \beta, \gamma}^O$. According to the equation, the total number of ${R}_{\alpha, \beta, \gamma}^O$ was 66, with 66 kinds of unique combinations of the three parameters.

The subjective method was expressed as Eq. (3).

3 $$ {R}_{w_i}^S=\frac{\sum_{p=1}^{30}{R}_{p,{w}_i}}{T_i} $$

where ${R}_{p,{w}_i}$represented the importance ranking of word *w*_*i*_ in sample *p* and was determined according to specific sample content. If word *w*_*i*_ did not appear in sample *p*, then ${R}_{p,{w}_i}$=0. *T*_*i*_ was the number of sample containing word *w*_*i.*_ ${R}_{w_i}^S$ was the average importance ranking of word *w*_*i*_.

The optimized combination of the three parameters was determined as Eq. (4).

4 $$ \Big\{{\displaystyle \begin{array}{c} del{t}_{\alpha, \beta, \gamma }={\sum}_{i=1}^n\mid {R}_{w_i}^S-{R}_{w_i,\alpha, \beta, \gamma}^O\mid \\ {} del{t}_{best}=\min \left( del{t}_{\alpha, \beta, \gamma}\right)\end{array}} $$

where *delt*_*α*, *β*, *γ*_was the sum of absolute values of the difference between ${R}_{w_i}^S$and ${R}_{w_i,\alpha, \beta, \gamma}^O$. *delt*_*best*_ was the minimum of *delt*_*α*, *β*, *γ*_.

Using the above method, we got the best combination with *α*=0.4, *β*=0.4, and *γ*=0.2.

### Affinity propagation clustering analysis

Affinity Propagation (AP) clustering algorithm based on message passing was proposed by Frey and Dueck [55]. Unlike clustering algorithms such as k-means or k-medoids, AP does not require the setting of cluster numbers in advance. Instead, it simultaneously considers all data points as potential exemplars and recursively transmits real-valued messages until a high-quality set of exemplars and corresponding clusters emerges [56]. For each node *i* and each candidate exemplar *k*, AP calculates the “responsibility” *r*(*i, k*) indicating the suitableness of *k* as an exemplar for *i*, and the “availability” *a*(*i, k*) reflecting the evidence that *i* should choose *k* as an exemplar.

5 $$ r\left(i,k\right)\leftarrow s\left(i,k\right)-\underset{k^{\prime }:{k}^{\prime}\ne k}{\max}\left\{a\left(i,{k}^{\prime}\right)+s\left(i,{k}^{\prime}\right)\right\} $$

6 $$ a\left(i,k\right)\leftarrow \min \left\{0,r\left(k,k\right)+\sum \limits_{i^{\prime }:{i}^{\prime}\notin \left\{i,k\right\}}\max \left\{0,r\left({i}^{\prime },k\right)\right\}\right\} $$

where the matrix *s*(*i, k*) indicates the similarities (e.g., edge weights) between two nodes *i* and *k*, and the diagonal of this matrix contains the preferences for each node. Equations (5) and (6) are iterated until a good set of exemplars emerges. Each node *i* can then be assigned to the exemplar *k* which maximizes the sum *a*(*i, k*) *+ r*(*i, k*). If *i = k*, then *i* is an exemplar. A damping factor between 0 and 1 is used to control numerical oscillations.

As reported in literature, AP achieves considerable improvement over standard clustering methods such as k-means [57], spectral clustering [58] and super-paramagnetic clustering [59]. It identifies clusters with lower error rate and lower time consumption [60].

We performed AP clustering using an R package *APCluster* [61] with a key terms correlation matrix as input data. The matrix was generated based on the co-occurrence matrix using Ochiai correlation coefficient calculated as ${O}_{ij}={A}_{ij}/\sqrt{A_i{A}_j}$. *A*_*i*_ *and A*_*j*_ represent the frequencies of key terms *W*_*i*_ and *W*_*j*_, respectively. *A*_*ij*_ donates the co-occurrence frequencies of *W*_*i*_ and *W*_*j.*_ The Ochiai coefficient is identical to cosine coefficient [62], thus it can express the similarity of two key terms in theme representation. The value range of *O*_*ij*_ is [0, 1]. The larger the *O*_*ij*_ is between two terms, the more similar the two terms are in theme representation.

## Results

### Literature distribution characteristics analysis

**Fig. 1:**
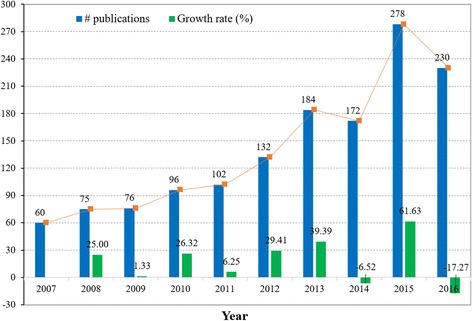
The number and growth rate of publications by year

The number and growth rate of publications by year are shown in Fig. 1. From the figure, the number of NLP-empowered medical research publications was overall showing an increasing trend. Until 2012 the number of publications was around 100 per year. From 2013 to 2015, the number of publications increased to around 200 per year. The annual growth rate reached 18.39% on average, while the rate reached up to 61.63% from 2014 to 2015, witnessing the research upsurge in 2015. According to regression analysis, the non-linear model with the smallest AIC and biggest $\overline{R^2}$ (Table 2) was selected out as *y* = 6.422397*10^6^–6.408129*10^6^x + 1.598485*10^6^ x^*2*^. With this model, the future research output can be estimated.

**Table 2:** AIC and $\overline{R^2}$of 3 fitting models

Model	AIC	$\overline{R^2}$
*y* = 0.06989x	117.1439	0.7829
*y* = −45,270.64+ 22.58x	98.70681	0.855
*y* = 6.422397*10^6^–6.408129*10^6^x + 1.598485*10^6^ x^2^	98.26147	0.8703

The 1405 publications were published in 324 unique sources. Table 3 shows the most productive 10 publication sources. These 10 sources together contributed more than 50% of the total publications. Among them, 8 belonged to journals, i.e., *Journal of the American Medical Informatics Association*, *Journal of Biomedical Informatics*, *BMC Bioinformatics*, *PloS ONE*, *Journal of Biomedical Semantics*, *Studies in Health Technology and Informatics*, *BMC Medical Informatics and Decision Making*, and *Biomedical Informatics Insights.* The rest 2 were conferences, i.e., *AMIA Annual Symposium Proceedings* and *AMIA Joint Summits on Translational Science Proceedings*.

**Table 3:** Top 10 most productive publication sources

Publication sources	# related pub.	Proportion of related pub. against 1405 pub. (%)	Total #pub. of the sources (Proportion of related pub. against total #pub.)
Journal of the American Medical Informatics Association	154	10.96	1689 (9.12%)
AMIA Annual Symposium Proceedings	153	10.89	2283 (6.70%)
Journal of Biomedical Informatics	133	9.47	1378 (9.65%)
Studies in Health Technology and Informatics	91	6.48	7434 (1.22%)
BMC Bioinformatics	61	4.34	6332 (0.96%)
PloS ONE	36	2.56	166,876 (0.02%)
AMIA Joint Summits on Translational Science Proceedings	32	2.28	331 (9.67%)
Journal of Biomedical Semantics	28	1.99	322 (8.70%)
BMC Medical Informatics and Decision Making	27	1.92	1071 (2.52%)
Biomedical Informatics Insights	22	1.57	59 (37.29%)
Total	737	52.46	N/A

There were 1386 publications with author affiliation information. The country distribution for first author affiliation was analyzed based on these publications. Table 4 shows the top 8 countries with the highest number of publications and GDP per capita. The USA and Australia were listed in top 8 for the two metrics. According to the Spearman’s rank correlation test applied to explore the relationship between publication numbers and GDP per capita, the testing *p*-value was 0.003, rejecting the null hypothesis at the significance level of 5%. And the Spearman’s rank correlation coefficient was 0.445.

**Table 4:** Publications and GDP per capita by country

Country	#pub.	Proportion	Country	GDP per capita (1000 US dollars)
United States	931	67.17%	Norway	897.046
United Kingdom	72	5.19%	Switzerland	780.731
China (including Hong Kong and Macao)	54	3.90%	Denmark	589.324
France	50	3.61%	Ireland	554.754
Canada	29	2.09%	**Australia**	551.685
Germany	28	2.02%	Sweden	545.730
Japan	24	1.73%	**United States**	514.139
Australia	23	1.66%	Netherlands	506.744

**Fig. 2:**
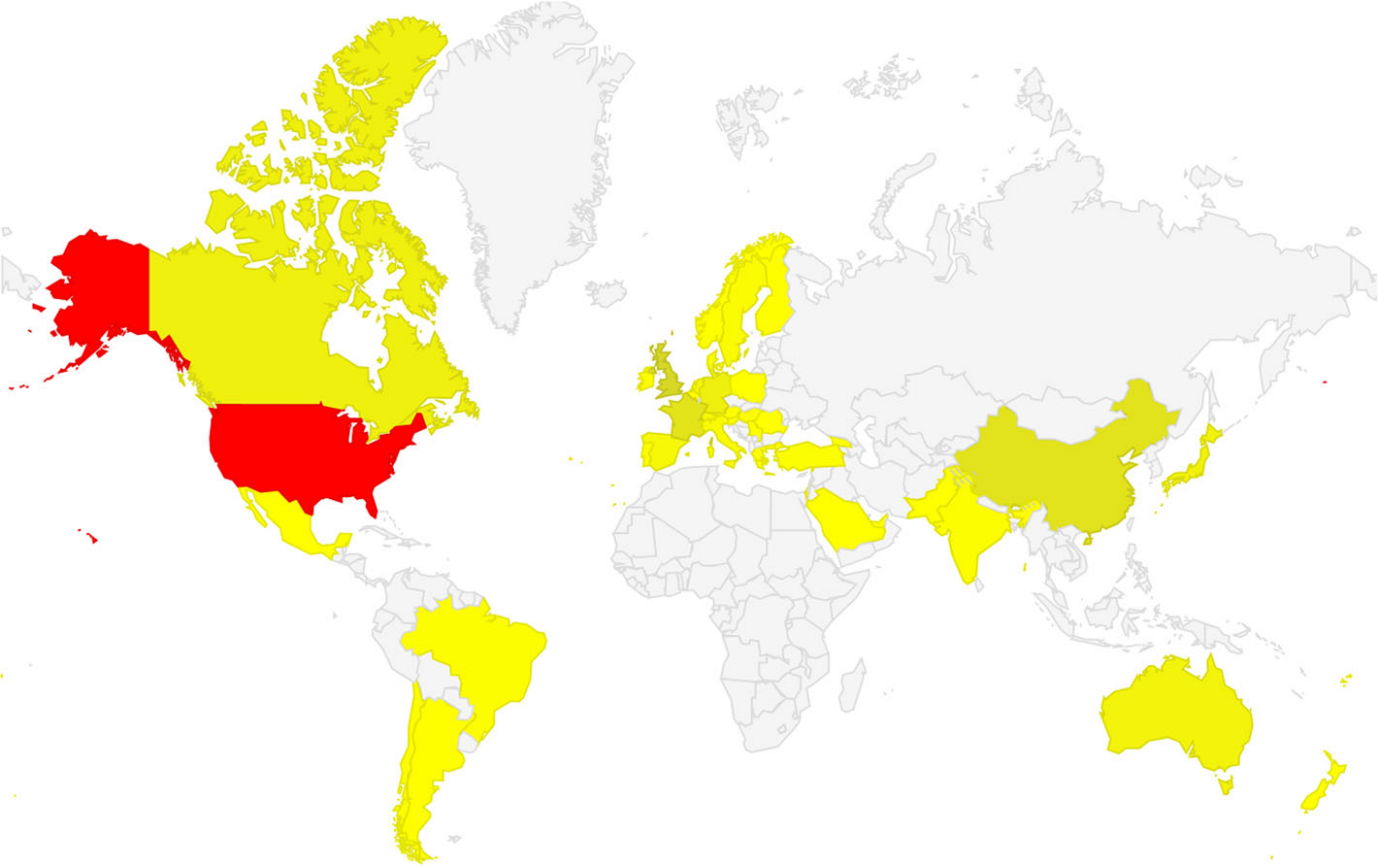
Geomap visualization of publications by country (the more publications one country had, the closer the color was to red)

Figure 2 is the Google geomap of country’s publications (access via the link [63]). A geomap is a map of a country or continent, with colors and values assigned to specific regions. Values are displayed as a color scale. Here the more publications one country had, the closer the color was to red. For the USA, the red region took a proportion up to 67.17%.

The top productive authors and first authors are presented in Table 5, where *Xu, Hua*, *Denny, Joshua C* and *Liu, Hongfang* were top 3 productive authors. The top 3 productive first authors were *Denny, Joshua C*, *Xu, Hua* and *Uzuner, Ozlem*. Three authors *Denny, Joshua C*, *Xu, Hua* and *Uzuner, Ozlem* appeared in both two ranks. The top productive author affiliations and first author affiliations are shown in Table 6, where *Mayo Clinic*, *The University of Utah*, and *Vanderbilt University* ranked top 3 in both ranks.

**Table 5:** Top productive authors and first authors

Rank	Authors	#pub.	Rank	First authors	#pub.
1	*Xu, Hua*	54	1	*Denny, Joshua C*	12
2	*Denny, Joshua C*	50	2	*Xu, Hua*	9
3	*Liu, Hongfang*	41	3	*Uzuner, Ozlem*	8
4	*Chute, Christopher G*	27	4	*Lacson, Ronilda*	7
5	*Chapman, Wendy W*	25	4	*Roberts, Kirk*	7
6	*Friedman, Carol*	24	6	*Deleger, Louise*	6
7	*Uzuner, Ozlem*	21	6	*Doan, Son*	6
8	*Savova, Guergana K*	20	6	*Fan, Jung-Wei*	6
9	*Solti, Imre*	19	6	*Gundlapalli, Adi V*	6
10	*Melton, Genevieve B*	18	6	*Meystre, Stephane M*	6
10	*Shen, Shuying*	18			
10	*Sohn, Sunghwan*	18

**Table 6:** Top productive author affiliations and first author affiliations

Rank	Author affiliations	#pub.	Rank	First author affiliations	#pub.
1	Mayo Clinic	86	1	Mayo Clinic	56
2	The University of Utah	82	2	The University of Utah	54
3	Vanderbilt University	78	3	Vanderbilt University	51
4	National Institutes of Health	64	4	Columbia University	43
5	Columbia University	59	5	National Institutes of Health	41
6	Brigham and Women’s Hospital	52	6	Brigham and Women’s Hospital	30
7	University of Washington	36	7	University of Minnesota	23
8	University of Pittsburgh	32	7	University of Pittsburgh	23
9	Massachusetts General Hospital	31	9	VA Salt Lake City Health Care System	21
9	Stanford University	31	10	Massachusetts General Hospital	19

### Scientific collaboration analysis

The result of publication cooperation analysis on the 1386 publications is shown in Table 7. The number of co-author publications was 1318 during the year 2007–2016 with an annual co-author rate around 90%. The co-affiliation rate was generally increasing. Until 2013 the co-affiliation rate was around 45% per year. From 2014 until 2016, the co-affiliation rate increased to above 60%. The annual co-country rate during 2007–2014 was between 6.38% and 13.33%, and the number reached up to around 16% in 2015 and 2016.

**Table 7:** The statistics of author and affiliation cooperation

Year	Total #pub.	#co-author pub.	Co-author rate%	#co-affiliation pub.	Co-affiliation rate%	#co-country pub.	Co-country rate%
2007	58	54	93.10	26	44.83	7	12.07
2008	73	64	87.67	32	43.84	8	10.96
2009	75	70	93.33	36	48.00	9	12.00
2010	94	85	90.43	44	46.81	6	6.38
2011	100	96	96.00	46	46.00	10	10.00
2012	129	121	93.80	63	48.84	13	10.08
2013	180	175	97.22	111	61.67	24	13.33
2014	171	161	94.15	111	64.91	22	12.87
2015	278	273	98.20	170	61.15	46	16.55
2016	228	219	96.05	146	64.04	36	15.79
Total	1386	1318	N/A	785	N/A	181	N/A

**Fig. 3:**
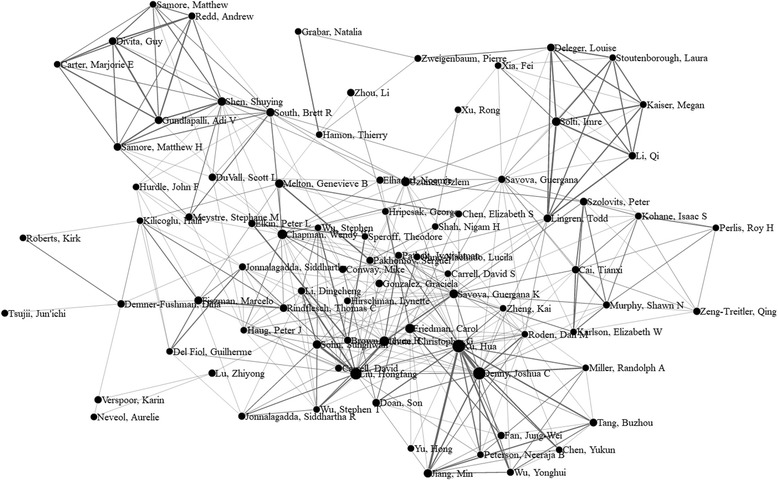
Force directed network of 87 authors with #pub. > = 8

**Fig. 4:**
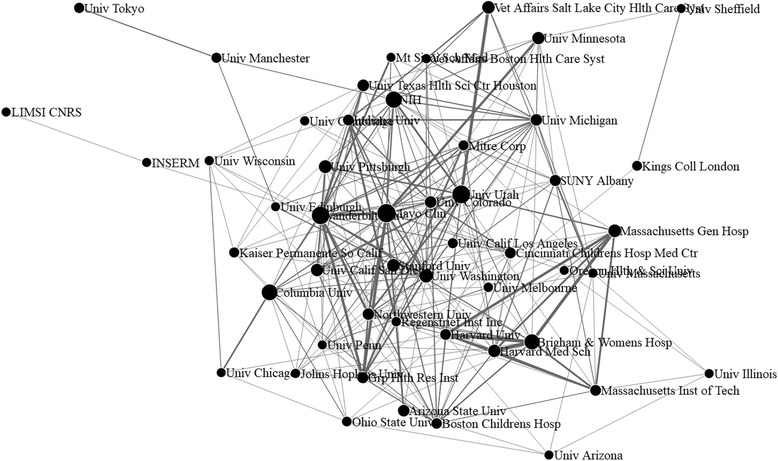
Force directed network of 50 affiliations with #pub. > = 10

We then visualized the cooperation among authors and affiliations. Fig. 3 is a generated network containing 87 authors with publications > = 8 (access via the link [64]). Fig. 4 shows a force directed network containing 50 affiliations with publications > = 10 (access via the link [65]). Furthermore, cooperation networks containing 204 authors with publications > = 5, and 108 affiliations with publications > = 5, as well as all authors and affiliations were also visualized (access via the link [66–69]). One can interactively drag and drop any node in the networks to view connections for any specific author or affiliation.

### Thematic discovery and evolution analysis

Using the optimized weights combination as *α*=0.4, *β*=0.4, and *γ*=0.2, we finally obtained top 50 key terms with highest frequencies. Based on these key terms, a 50*50 co-occurrence matrix was generated, where the top 10 are shown in Table 8. The values on the main diagonal of the matrix donated the frequencies of terms and the values on the non-main diagonal indicated the numbers of publications that two terms appeared together.

**Table 8:** The top 10 key terms in the co-occurrence matrix

	Artificial intelligence	Data mining	Electronic health record	Female	Information storage and retrieval	Machine learning	Medical record	Patient	Precision	Semantics
Artificial intelligence	185	52	53	11	56	40	25	33	40	33
Data mining	52	288	122	31	20	53	38	55	46	52
Electronic health record	53	122	420	78	80	60	95	167	77	40
Female	11	31	78	169	15	10	46	82	18	10
Information storage and retrieval	56	20	80	15	239	18	30	42	47	47
Machine learning	40	53	60	10	18	162	25	39	30	22
Medical record	25	38	95	46	30	25	178	77	29	8
Patient	33	55	167	82	42	39	77	326	59	19
Precision	40	46	77	18	47	30	29	59	217	34
Semantics	33	52	40	10	47	22	8	19	34	165

**Fig. 5:**
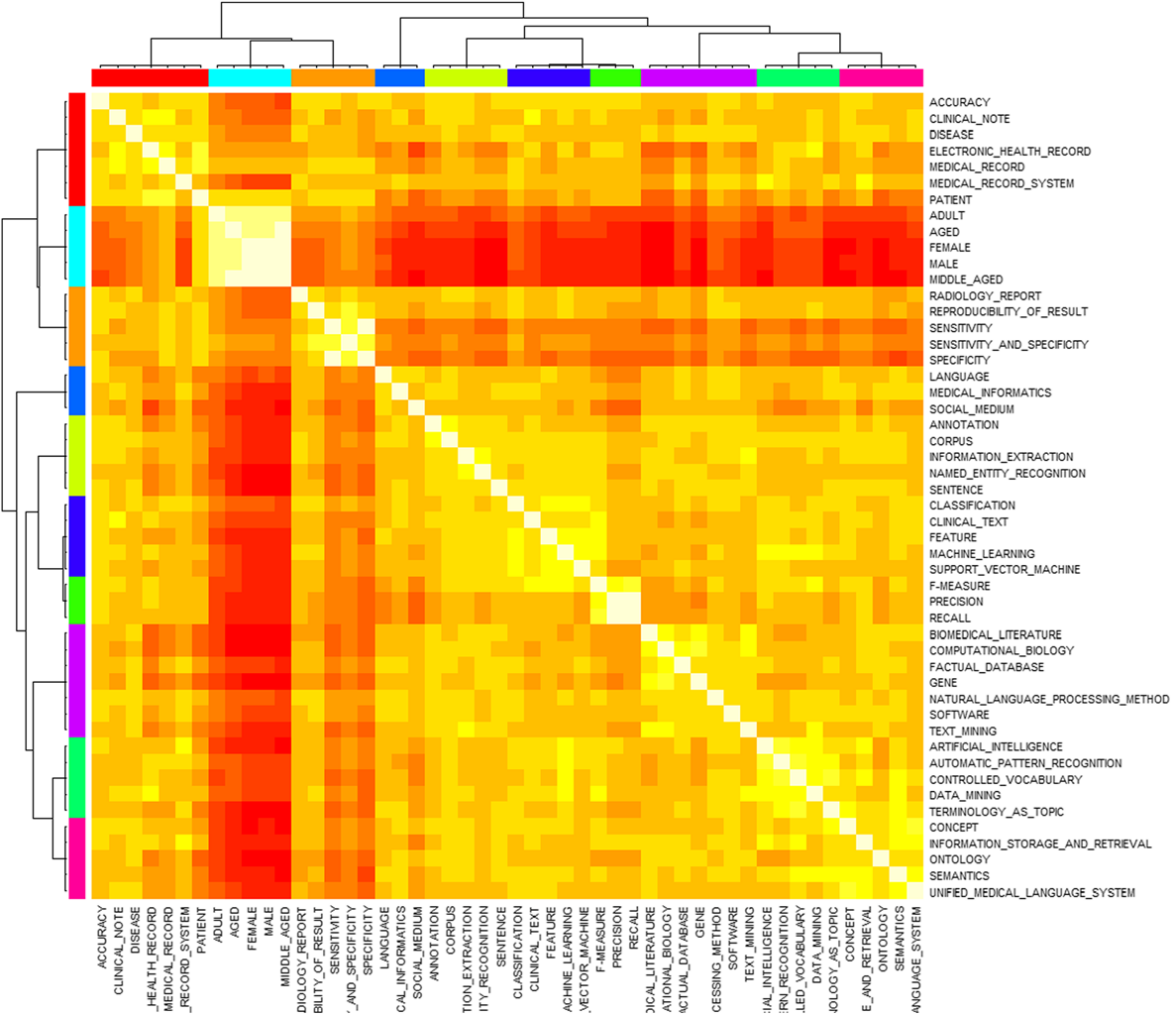
Heatmap of AP clustering result for the 2007–2016 period

The correlation matrix generated using Ochiai correlation coefficient was then used for AP clustering. The clustering result for the publication during the year 2007–2016 was as Fig. 5 and Table 9. The top 50 key terms were distributed into 10 clusters. We manually labelled each cluster by analyzing the meaning of representative terms and reviewing abstract content.

**Table 9:** AP clustering result for the publication during the year2007–2016

Cluster	Theme	Key terms
1	Computational biology	**Computational biology**; Biomedical literature; Factual database; Gene; Natural language processing method; Software; Text mining
2	Terminology mining	**Controlled vocabulary**; Artificial intelligence; Automatic pattern recognition; Data mining; Terminology as topic
3	Information extraction	**Corpus**; Annotation; Information extraction; Named entity recognition; Sentence
4	Text classification	**Feature**; Classification; Clinical text; Machine learning; Support vector machine
5	Social medium as data source	**Language**; Medical informatics; Social medium
6	Clinical information	**Medical record**; Accuracy; Clinical note; Disease; Electronic health record; Medical record system; Patient
7	Patient characteristics	**Middle aged**; Adult; Aged; Female; Male
8	Performance measurements	**Recall**; F-measure; Precision
9	Outcome evaluation	**Sensitivity and specificity**; Radiology report; Reproducibility of result; Sensitivity; Specificity
10	Information retrieval	**Unified medical language system**; Concept; Information storage and retrieval; Ontology; Semantics

We further compared theme distribution for periods 2007–2011 and 2012–2016, their AP clustering results is shown in Table 10. As for the two periods, top 50 terms were clustered into 12 clusters. Clusters with same exemplars (i.e., cluster 1–8) were placed in top rows. Terms in bold type donated newly emerging terms for 2012–2016 period comparing with 2007–2011.

**Table 10:** Comparison of AP clustering results for the 2007–2011 and 2012–2016 periods

Cluster	2007–2011	Cluster	2012–2016
1	Text mining; Abstracting and indexing as topic; Annotation; Database management system; Sentence	1	Text mining; **Information extraction**; **Named entity recognition**
2	Female; Male	2	Female; Male; **Middle aged**; **Adult**; **Aged**
3	Recall; Precision; F-measure	3	Recall; Precision; F-measure; Accuracy
4	Artificial intelligence; Information storage and retrieval; Automatic pattern recognition	4	Artificial intelligence; Semantics; Information storage and retrieval; Clinical text; Concept; Language; Sentence; Unified medical language system
5	Computational biology; Factual database; Gene; Protein; Protein-protein interaction	5	Computational biology; Factual database; Software; **Free text**
6	Classification; Feature; Semantics; Data mining; Natural language processing method; Unified medical language system	6	Classification; Feature; Support vector machine; **Classifier**
7	Patient; Disease; Medical record; Medical record system; Patient discharge; Sensitivity and specificity	7	Patient; Medical record; Electronic health record; Clinical note
8	Medical informatics; User-computer interface; Software	8	Medical informatics; Annotation; Corpus; Gene; **Social medium**
9	Clinical text; Accuracy; Clinical decision support system; Clinical note; Electronic health record; Natural language processing system; Support vector machine	9	Automatic pattern recognition; Controlled vocabulary; Data mining; **Machine learning**
10	Word; Corpus; Language	10	**Sensitivity**; **Confidence interval**; **Specificity**
11	Biomedical literature; Knowledge; Medline; Ontology	11	**Reproducibility of result**; **Radiology report**; Sensitivity and specificity
12	Terminology as topic; Concept; Controlled vocabulary	12	Disease; Natural language processing method; **Phenotype**

First term in each cluster donates exemplar. Terms in bold type donate new emergent terms for 2012–2016 period compared with 2007–2011 period

## Discussion

A bibliometric analysis of NLP-empowered medical research publications from PubMed during the year 2007–2016 has been conducted. The analysis included three aspects: literature distribution characteristics analysis, scientific collaboration analysis, and thematic discovery and evolution analysis. Some findings were as follows:

1. The NLP-empowered medical research field has attracted the interests of scientific research community throughout years, which was observed in the annual growth of publications.
2. 10 most productive publication sources together contributed more than 50% of the 1405 publications. The top 3 were: *Journal of the American Medical Informatics Association*, *AMIA Annual Symposium Proceedings*, and *Journal of Biomedical Informatics*.
3. The USA had the highest number of publications with a proportion up to 67.17%. A moderately significant correlation between country’s publications and GDP per capita was revealed by the Spearman’s rank correlation coefficient as 0.445.
4. We have identified prominent authors that have made significant contributions to the research field. Top productive authors included *Denny, Joshua C*, *Xu, Hua*, *Uzuner, Ozlem*, and *Liu, Hongfang*.
5. The top 3 most productive affiliations including *Mayo Clinic*, *The University of Utah*, and *Vanderbilt University* have devoted 17.75% of the 1386 publications.
6. The annual co-affiliation rate increased to above 60% from 2014 until 2016, and the annual co-country rate reached up to around 16% in 2015 and 2016. The cooperation among specific authors and affiliations were visualized using network graphs.
7. The NLP-empowered medical research focused on 10 main thematic areas during the year 2007–2016 including *Computational biology*, *Terminology mining*, *Information extraction*, *Text classification*, *Social medium as data source*, *Clinical information*, *Patient characteristics*, *Performance measurements*, *Outcome evaluation*, and *Information retrieval*.
8. By observing the newly emerging terms in Table 10, some differences and new research topics can be identified for recent research during the year 2012–2016 compared with 2007–2011. Especially, cluster 1, 2, 8, 9, 10, 11, and 12 were ought to be paid attention to. For example, *Information extraction* and *Named entity recognition* have become more popular in medical research during the year 2012–2016. For Cluster 2, terms related to age, i.e., *Middle aged*, *Adult*, and *Aged*, indicated that researchers gradually paid more attention to the age characteristic of target population in addition to gender. In cluster 8 for 2012–2016 period, the new term *Social medium* indicated a focus on utilizing social media data for medical analysis [70]. The new term *Machine learning* in cluster 9 for 2012–2016 period witnessed increasing interest in combining *Machine learning* and NLP techniques, e.g., [71, 72].

The findings can potentially benefit relevant researchers, especially newcomers in: understanding the research performance and recent development of NLP-empowered medical research field, selecting scientific cooperation partners with the knowledge of predominant authors, affiliations, and countries, optimizing research topic decision to keep abreast of current research hotspots, and monitoring new scientific or technological activities.

In term importance weighting, the combination of subjective and objective methods was used. The subjective weighing result might vary from person to person due to subjective judgment. Thus, in this paper, we ranked the importance by semantics analysis as well as reviewing text content to keep high consistence with text intention.

In our study, AP clustering method was performed based on top 50 high frequency key terms in order to acquire a moderate number of categories. However, this might result in the ignorance of some sudden terms that are possible for representing research fronts although with low frequencies. Therefore, in our future work, we will make improvement by trying alternative methods such as Latent Dirichlet Allocation to consider every single term.

The AP clustering results were on the whole reasonable and easy-to-understand. However, we still found that terms with similar semantics, i.e., Data mining and Text mining were not clustered into the same cluster in the same context NLP, which might cause confusion. AP clustering was conducted based on the key terms correlation matrix, and the matrix was calculated using Ochiai correlation coefficient. Hence, the clustering results might be vulnerable to choices of both calculation method and clustering method. Therefore, in our future work, we will conduct comparison on different calculation methods of correlation matrix as well as different clustering methods for further exploration.

In the study, PubMed as the biggest medical related publication resource was used as data source. However, a minor number of publications might be in NLP-related journals and conferences. Thus, in our future work, we will consider including these journals and conference as additional publication sources.

## Conclusions

This paper presents a bibliometric analysis of NLP-empowered medical research publications from PubMed during the year 2007–2016 with the purpose of understanding the research status of the field. Some literature distribution characteristics including productive publication sources, authors, affiliations, and countries are provided with statistics analysis methods. Scientific collaborations among authors and affiliations are visualized with network analysis method. Affinity propagation clustering method is used for thematic discovery and evolution analysis. Some interesting results and findings are presented. To our knowledge, there was no similar study thoroughly examining NLP-empowered medical research publications. Our work can potentially assist relevant researchers, especially newcomers in keeping abreast of the NLP-empowered medical research status, seeking scientific cooperation partners, optimizing research topics choices, and monitoring new scientific or techno-logical activities.

## Abbreviations

AIC: Akaike Information Criterion
AP: Affinity Propagation
EHR: Electronic Health Record
EHRs: Electronic Health Records
EMRs: Electronic Medical Records
GDP: Gross Domestic Product
MeSH: Medical subject headings
NIH: National Institutes of Health
NLP: Natural Language Processing
USA: United States
WoS: Web of Science
